# Iatrogenic Acute Ascending Aortic Dissection with Intramural Hematoma during Coronary Artery Stenting: A Case Report

**DOI:** 10.3389/fsurg.2017.00002

**Published:** 2017-02-03

**Authors:** Mohamad El-Haress, Hicham Daadaa, Shima Shahjouei, Firas El-Bitar, Hisham Bahmad

**Affiliations:** ^1^Faculty of Medicine, Beirut Arab University, Beirut, Lebanon; ^2^Department of Neurosurgery, Children’s Hospital Medical Center, Tehran University of Medical Sciences, Tehran, Iran; ^3^Department of Cardiothoracic Surgery, Hammoud Hospital University Medical Center, Saida, Lebanon

**Keywords:** acute aortic dissection, intramural hematoma, percutaneous coronary intervention, coronary artery bypass graft, case report

## Abstract

**Background:**

Iatrogenic acute ascending aortic dissection during percutaneous coronary intervention (PCI) is an exceptionally rare and life-threatening sequel that requires early and accurate diagnosis along with rapid management. No guidelines have yet been established to direct decisions on the different treatment options that can be employed in the setting of acute aortic dissections caused by PCI. However, similar cases have been treated either by intracoronary stenting and conservative management as in localized aortocoronary dissections or by surgical intervention in cases of extensive aortic dissections.

**Case summary:**

Hereby, we present a rare case of a female patient who developed “full-blown” acute ascending aortic dissection (Stanford type A—DeBakey type II dissection) with intramural hematoma during an elective percutaneous transluminal coronary angioplasty (PTCA) of the right coronary artery (RCA) and left circumflex artery (LCA). Accordingly, emergent surgical repair of the dissected aorta was performed including grafting of supracoronary ascending aortic tube, along with coronary artery bypass graft placement and septal myomectomy for severely hypertrophied cardiac septum. The patient recovered successfully without any documented postoperative complications.

**Conclusion:**

It is pivotal to avoid aggressive use of instrumentation during PTCA in order to prevent the potential development of catheter-induced aortic dissection.

## Introduction

Acute aortic dissection following percutaneous coronary intervention (PCI) is an extremely rare and potentially fatal event (1). This life-threatening complication, which may occur due to aggressive manipulation during percutaneous transluminal coronary angioplasty (PTCA), necessitates early and rapid diagnosis along with emergent treatment. Treatment options vary from conservative management (2) to invasive aortic dissection surgical repair and revascularization (3). The prevalence of acute aortic dissections following PCI is unknown exactly; however, the first case was reported in the year 1992 (4). Recently in February 2016, Shah et al. reported two new case reports of aortic dissections caused by PCI with detailed analysis of the 86 previously reported cases worldwide (5).

**Figure 1:**
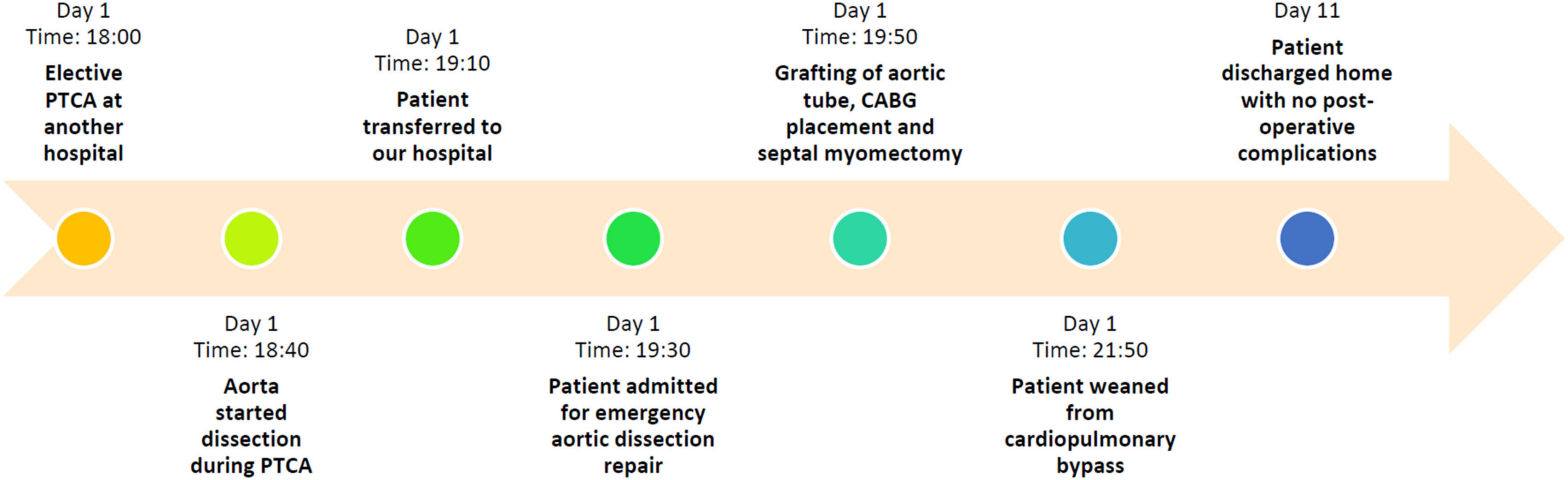
**Timeline summarizing major events of the case**.

This rare type of acute aortic artery dissection usually occurs as a complication in patients with a history of long-standing hypertension or cystic medial necrosis of the aortic wall (6). Other common complications of PCI include coronary artery dissection, which can be treated by intracoronary stenting (6), cardiac tamponade (7), and coronary artery intramural hematoma (8). Options of treatment are determined after assessing the patient’s stability and knowing the nature of dissection and its extent, knowing that no guidelines have yet been established to address this point specifically. Hereby, we present a rare case of a female patient who developed an iatrogenic acute “full-blown” aortic dissection with intramural hematoma during elective PTCA (Figure 1). This case report was conducted and reported in accordance with CAse REports (CARE) guidelines for reporting case reports (supplement 1).

## Case Presentation

A 75-year-old female was transferred from another hospital to the emergency department of our hospital for acute aortic dissection during coronary angioplasty. She had a history of dyslipidemia, diabetes mellitus type 2, and hypertension, all diagnosed 5 years ago. She also had a history of urinary tract infections. Her surgical history was insignificant. She had no known food or drug allergies. Home medications included aspirin 100 mg (Bayer Bitterfeld GmbH, Germany), lovenox 4,000 IU anti-Xa/0.4 mL (Sanofi Winthrop Industrie, France), plavix 75 mg (Sanofi Pharma Bristol Myers Squibb SNC, France), concor 5 mg (Merck KGaA, Germany), and glucophage 850 mg (Merck Santé SAS, France). She had 13 pregnancies and 9 full-term births by normal vaginal deliveries with no complications. No history of cigarette smoking, drug abuse, alcohol intake, or multiple sexual partners was reported.

Prior to presentation to our hospital, patient had been undergoing an elective PTCA at another hospital for acute ST-elevation myocardial infarction, when iatrogenic acute ascending aortic dissection occurred. During PTCA, an MP 3.75 4Fr guide catheter had been used to perform the coronary angiography, revealing double vessel coronary artery disease with a 90% proximal left circumflex artery (LCX) lesion and 90% right coronary artery (RCA) lesion. While inserting the Launcher 6Fr 3.75 guide catheter, resistance occurred with the brachiocephalic artery due to calcified aortic arch. Reperfusion was successfully achieved afterward by passing an ORSIRO sirolimus-eluting coronary stent (Biotronik, Bülach, Switzerland) into the RCA. However, while inserting the second stent into the LCX, aorta started its dissection. Therefore, the patient was transferred urgently to our hospital within 30 min for coronary artery bypass graft (CABG) and aortic dissection repair. Fortunately, the patient remained hemodynamically stable throughout.

At presentation to the emergency department of our hospital, the patient was sedated, afebrile (36.8°C) with blood pressure of 150/100 mmHg, pulse rate of 68 beats per minute, a respiratory rate of 18 breaths per minute, and O_2_ saturation of 99%. Physical examination was normal except for bruit heard at the aortic area, with regular first and second heart sounds and no murmurs. On electrocardiogram, there was atrial fibrillation and rapid ventricular response with aberrant conduction or ventricular premature complexes. Emergent trans-thoracic echography was performed revealing an estimated ejection fraction of 60% and enlarged ascending aorta. Patient was admitted for emergency CABG surgery and aortic dissection repair.

**Figure 2:**
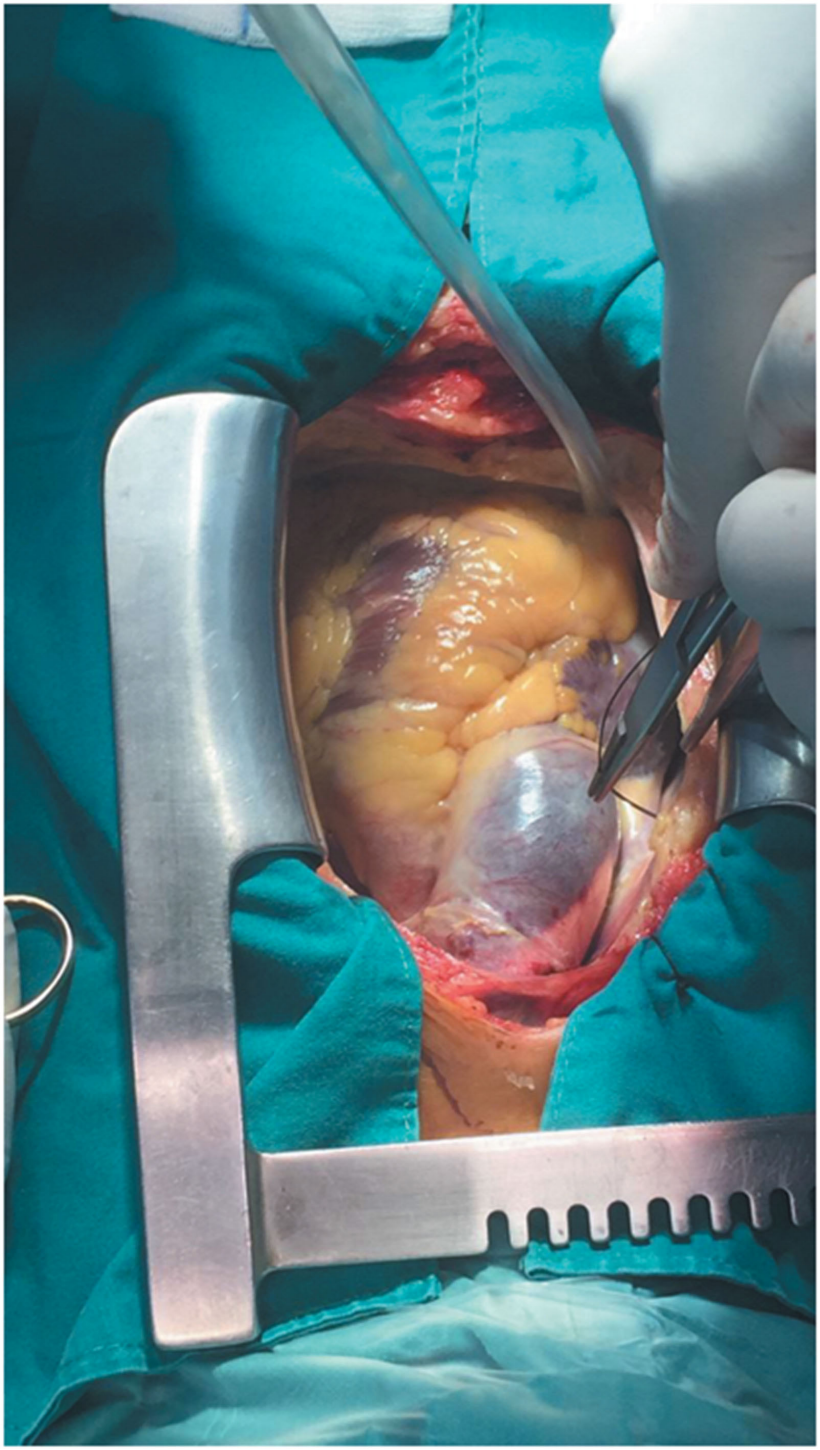
**Stanford type A DeBakey type II aortic dissection showing enlarged ascending aorta with an approximate diameter of 4.5 cm and dark reddish in color along its length**. Extent of the aortic dissection reaches nearly 7 cm high.

**Figure 3:**
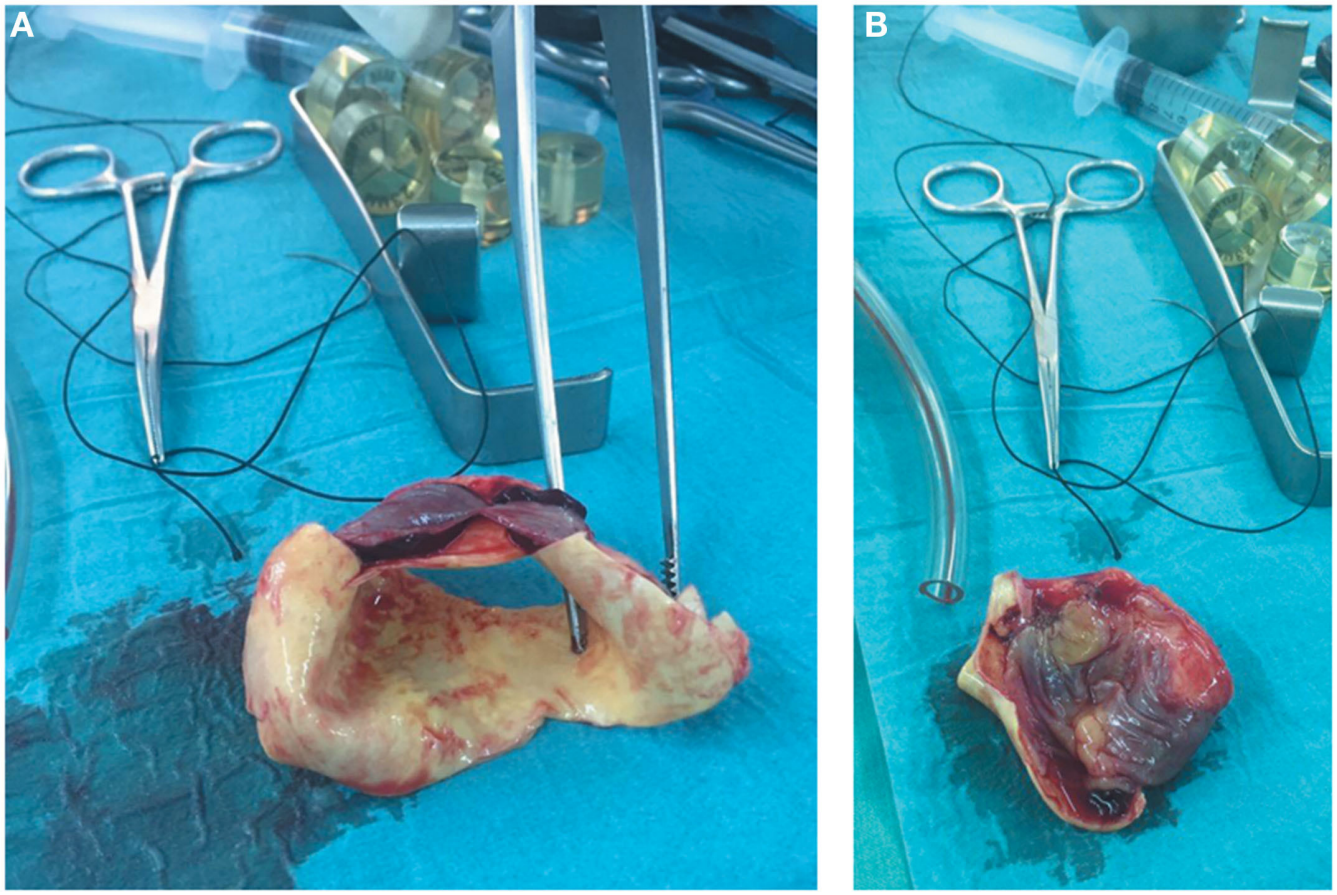
**(A)** Section of the dissected ascending aorta showing the intramural hematoma that was found behind the brachiocephalic trunk. **(B)** Section of the dissected ascending aorta revealing calcified left coronary cusp of the aortic valve and dissection entry, measuring around 5 mm, which was situated at the intima of the anterior wall of the ascending aorta, near the sinotubular junction.

**Figure 4:**
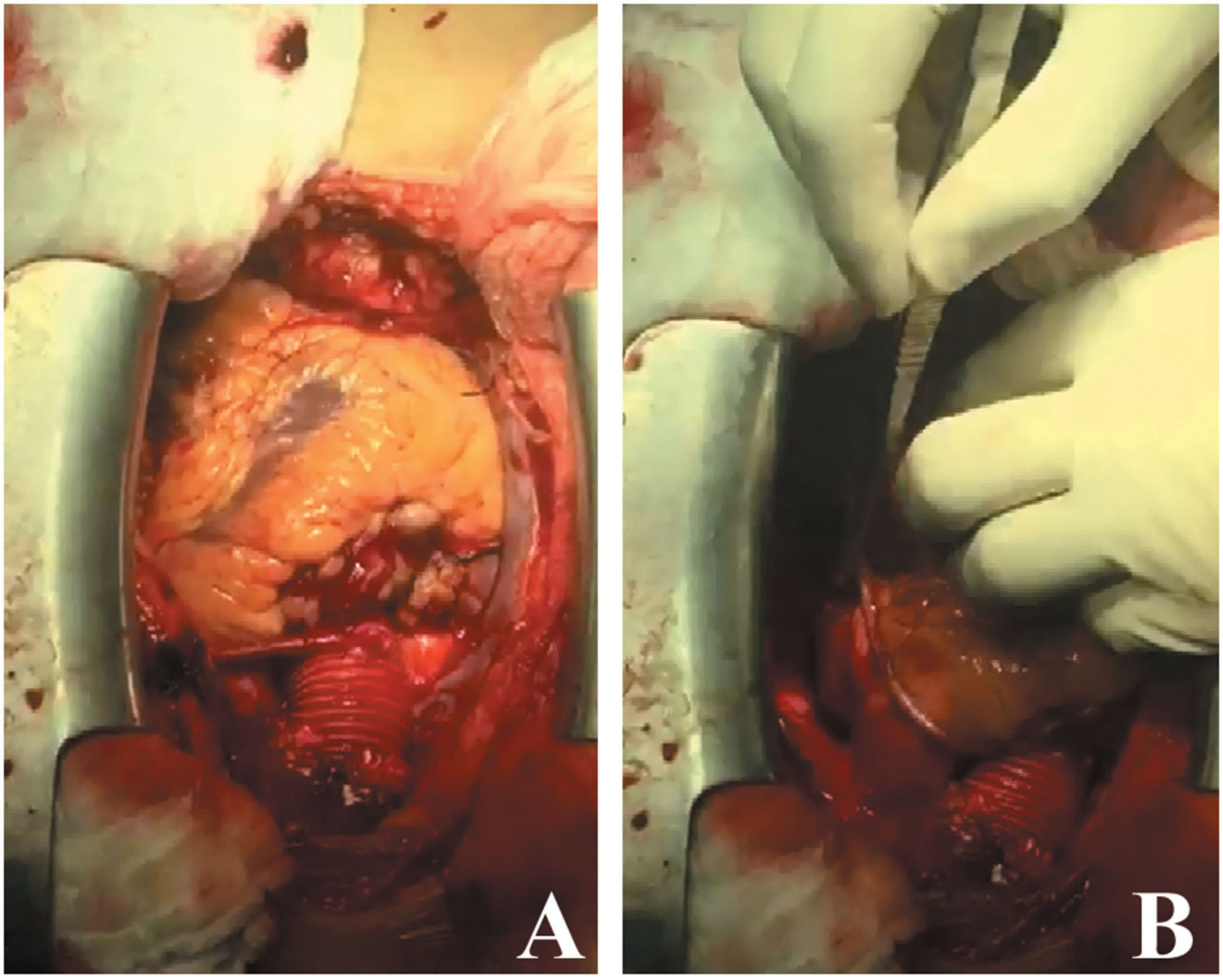
**(A)** Supracoronary aortic tube graft 28 mm, 7 cm in length, placed directly at the sinotubular junction. **(B)** Saphenous vein graft taken from the left lower extremity placed between the grafted ascending aorta and the LCX.

During CABG surgery, Stanford type A DeBakey type II ascending aortic dissection with intramural hematoma behind brachiocephalic trunk was found. The ascending aorta was enlarged with an approximate diameter of 4.5 cm and dark reddish in color along its length (Figure 2). It was opened after hypothermic circulatory arrest induction at 22°C, revealing calcified left coronary cusp of the aortic valve (Figures 3A,B). The dissection entry, measuring around 5 mm, was situated at the intima of the anterior wall of the ascending aorta, near the sinotubular junction. Extent of the aortic dissection reaches nearly 7 cm high. First, decalcification of the aortic valve cusp was done, and then due to the presence of 25 mm hypertrophy of the cardiac septum, 1 cm septal myomectomy was performed. After that, 7 cm length supracoronary aortic tube graft 28 mm (InterVascular Inc., LaCiotat, France) was placed directly at the sinotubular junction (Figure 4A). Saphenous vein graft from the left lower extremity was also placed between the grafted ascending aorta and the LCX (Figure 4B). The patient was successfully weaned from cardiopulmonary bypass and discharged after recovery on the 11th postoperative day. No postoperative complications were reported afterward.

## Discussion

Acute aortic dissection during PCI remains a very rare complication, with an overall incidence of 0.02% (9). Most previously reported cases state that the entry point of the dissection is located within a dissected coronary artery, with few studies reporting isolated ascending aortic dissection without coronary arteries being involved (10). In our case, the dissection entry was situated at the intima of the anterior wall of the ascending aorta, near the sinotubular junction, and not involving the coronary arteries.

Several proposed mechanisms have been described in literature to explain the etiology of this fatal event, including retrograde spread of a coronary artery dissection as a result of mechanical trauma by instruments used during the procedure, such as a guide catheter, wire, inflated balloon, etc. (5). Severely calcified aorta has also been suggested to be a risk factor for development of this complication due to the need of aggressive guiding catheter manipulation for insertion of coronary balloons and stents (2).

In our patient, we assume that multiple causes contributed to the development of ascending aortic dissection with hematoma. First, the calcified aortic arch may explain the use of aggressive manipulation of the guiding catheter during PTCA for stent insertion in the RCA and LCX. Besides, we postulate that this aggressive handling during PTCA may have occurred because of the severely calcified left coronary cusp of the aortic valve, especially that the intimal tear was found to be located at the anterior wall of the ascending aorta, near the sinotubular junction.

Considering management of such cases, Dunning et al. categorized aortocoronary dissection according to the level of aortic involvement, where class I denotes dissection involving only the coronaries, class II extending up to <40 mm of the ascending aorta, and class III reaching >40 mm of the ascending aorta (11). As class I and II patients with limited involvement of the aorta can benefit from stenting of the coronary dissection entry point without the need for surgical intervention, it was found that urgent surgery is the treatment of choice for class III patients with extensive dissection or patients with hemodynamic instability and those with ischemia of one of the aortic branches (11). In our patient, the iatrogenic “full-blown” ascending aortic dissection occurred during elective PTCA and was extensive reaching >40 mm. The patient as well experienced hemodynamic deterioration because of the hematoma, which was forming behind the trunk of the brachiocephalic artery. Therefore, emergent CABG surgery for aortic dissection repair was required despite the fact that the patient was on antiplatelet therapy prior to the surgery, which puts her at high risk of bleeding during or after the procedure.

## Concluding Remarks

Our present case discloses an iatrogenic acute “full-blown” ascending aortic dissection without coronary artery involvement, associated with intramural hematoma in the aorta behind the brachiocephalic trunk, as a complication of aggressive manipulation during PTCA and guide catheter use. The patient recovered uneventfully after emergency grafting of supracoronary aortic tube. However, due to the extremely dangerous and life-threatening nature of this complication, it is pivotal to avoid aggressive use of instrumentation during PTCA in order to prevent the potential development of catheter-induced aortic dissection.

## Ethics Statement

Written informed consent was obtained from the patient for publication of this case report and accompanying images. A copy of the written consent is available upon request for review by the Editor-in-Chief of this journal.

## Author Contributions

ME-H was responsible for getting the clinical data from medical records of the hospital and writing Section “Introduction.” HD and FE-B were responsible for writing the case presented. FE-B and SS provided other authors with explanations about the case reported. HB was responsible for writing the discussion and editing the whole manuscript, in addition to proofreading.

## Conflict of Interest Statement

The authors declare that the research was conducted in the absence of any commercial or financial relationships that could be construed as a potential conflict of interest. The reviewer JR and handling Editor declared their shared affiliation, and the handling Editor states that the process nevertheless met the standards of a fair and objective review.
