# Supplementary material for: Iatrogenic Acute Ascending Aortic Dissection with Intramural Hematoma during Coronary Artery Stenting: A Case Report
Source: Front Surg. 2017 Feb 3;4:2. doi: 10.3389/fsurg.2017.00002 (PMC5290308; doi:10.3389/fsurg.2017.00002)
Supplement: Supplementary file 1 [file Image_1.PDF]

CARE Checklist – 2016: Information for writing a case report

Topic	Item	Checklist item description	Line/Page
Title	1	The words "case report" should be in the title along with the area of focus	Line 3 / Page 1
Key Words	2	Four to seven key words—include "case report" as one of the key words	Lines 33-34 / Page 1
Abstract	3a	Background: What does this case report add to the medical literature?	Lines 3-10 / Page 2
	3b	Case summary: chief complaint, diagnoses, interventions, and outcomes	Lines 10-18 / Page 2
	3c	Conclusion: What is the main "take-away" lesson from this case?	Lines 18-20 / Page 2
Introduction	4	The current standard of care and contributions of this case—with references (1-2 paragraphs)	Lines 1-24 / Page 3
Timeline	5	Information from this case report organized into a timeline (table or figure)	Page 3 / Fig.1
Patient Information	6a	De-identified demographic and other patient or client specific information	Lines 28-38 / Page 3
	6b	Chief complaint—what prompted this visit?	Lines 40-43 / Page 3
	6c	Relevant history including past interventions and outcomes	Lines 43-50 / Page 3
Physical Exam	7	Relevant physical examination findings	Lines 5-9 / Page 4
Diagnostic Assessment	8a	Evaluations such as surveys, laboratory testing, imaging, etc.	Lines 9-13 / Page 4
	8b	Diagnostic reasoning including other diagnoses considered and challenges	Lines 12-13 / Page 4
	8c	Consider tables or figures linking assessment, diagnoses and interventions	Page 4 / Fig. 2, 3, 4
	8d	Prognostic characteristics where applicable	Not Applicable
Interventions	9a	Types such as life-style recommendations, treatments, medications, surgery	Lines 15-27 / Page 4
	9b	Intervention administration such as dosage, frequency and duration	Not Applicable
	9c	Note changes in intervention with explanation	Not Applicable
	9d	Other concurrent interventions	Lines 15-27 / Page 4
Follow-up and Outcomes	10a	Clinician assessment (and patient or client assessed outcomes when appropriate)	Lines 27-29 / Page 4
	10b	Important follow-up diagnostic evaluations	Not Applicable
	10c	Assessment of intervention adherence and tolerability, including adverse events	Lines 27-29 / Page 4
Discussion	11a	Strengths and limitations in your approach to this case	Discussion / Page 5
	11b	Specify how this case report informs practice or Clinical Practice Guidelines (CPG)	Lines 6-20 / Page 5
	11c	How does this case report suggest a testable hypothesis?	Lines 48-50 / Page 4<br>Lines 1-4 / Page 5
	11d	Conclusions and rationale	Lines 24-31 / Page 5
Patient Perspective	12	When appropriate include the assessment of the patient or client on this episode of care	Lines 6-20 / Page 5
Informed Consent	13	Informed consent from the person who is the subject of this case report is required by most journals	Lines 1-5 / Page 6
Additional Information	14	Acknowledgement section; Competing Interests; IRB approval when required	Lines 11-21 / Page 6
